# The Emerging Vision of the *JACMP* – Part 1

**DOI:** 10.1120/jacmp.v14i2.4373

**Published:** 2013-03-04

Throughout its 13 years of experience, the *JACMP* was a continual work in progress as an open‐source, open‐access academic journal. We have discovered there are both benefits and challenges to providing articles without cost to the world. The benefits are mostly obvious: immediate and free access to all articles, wider distribution of academic knowledge, ever‐growing participation by medical physicists throughout the planet, and the consequent benefit to the tens of millions of patients that undergo radiation oncology and imaging procedures each year. The primary challenge is this: The more “successful” any open‐access journal becomes, the more articles are submitted, accepted, and published. Therefore costs rise and donated time becomes burdensome, while at the same time, there is only a limited and finite amount of revenue that may be expected from banner ads. So we often return to and test our first principles of why the *JACMP* was needed and what it means now.

When I turned the *JACMP* over to George Starkschall five years ago, I was seeing about 40 articles under review and between 12 and 25 articles in editing, depending on where we happened to be in the quarterly publishing cycle. These numbers have about tripled in the intervening years. Today, I see about 120 articles under review, and between 35 and 60 articles in editing, depending on the now bimonthly publishing cycle. The time and effort of the Editor‐in‐Chief is directly proportional to the number of articles. I find that despite a recent decision by the AAPM Board of Directors to compensate the *JACMP* Editor‐in‐Chief, I am actually donating more time to the *JACMP* than I was when I surrendered the Journal to George in January of 2008.

That the *JACMP* has established a premier international presence is due entirely to the tireless, thorough, efficient, and politically savvy editing efforts of George Starkschall. Each day, the *JACMP* website is accessed by over a thousand unique IP addresses, and each month by almost 10,000. There are a total of approximately 4,500 registered users of the *JACMP* which includes reviewers, authors, and editors. George has moved the bar far higher and it will be a challenge, both for me and for any editor who follows, to maintain the level of excellence he demonstrated as Editor‐in‐Chief. So George, let me document sincere thanks and admiration not just for myself, but on behalf of all of the Section Editors, Reviewers, and Authors for what you did for the *JACMP*. Thank you for the thousands of hours you donated to make the *JACMP* what it has become.

This is a lesson for the younger medical physicists. While few have sacrificed as much as George, many medical physicists devote hundreds of hours each year in volunteer efforts to our profession. Are you doing your part?

## THE MORAL IMPERATIVE

In 1998, many clinical physicists were puzzled because there were limited options available to publish clinical articles and make them available to the clinical physicist community. It seemed wrong to put barriers in the way of articles that would help clinical physicists do their work better. It seemed equally wrong for patients not to have the benefit of their medical physicists being able to access the best and most contemporary clinical information. The Internet, which was relatively new at the time, offered hope.

Yes, *Medical Physics* was publishing outstanding scientific articles, and I was grateful to receive it each month. It was well worth the cost of the subscription, which I was (and am) required to pay as an AAPM member. But while we all valued the scientific articles, it seemed there was a community consensus to ascribe less value to clinical contributions. Perhaps if the *JACMP* offered the articles for free (at least for a season), no one would protest.

The big question that could only be answered over a number of years is: What is the value of clinical (or for that matter nonclinical) academic articles that are offered for free? What we are finding is that the current medical physics generation both appreciates and largely expects academic articles to be available without cost or barriers. This is not to say these articles are not valuable and worthwhile. I believe it can now be maintained that the intrinsic value of open‐access medical physics articles is now an established fact, worldwide. Most universities now require that open‐access articles be valued equally with those published in traditional journals when considering promotion and tenure for faculty. Additionally, the value of open‐access academic publishing now extends far beyond medical physics, radiology, and medicine to the entire academic community, as well. The *JACMP* was a very early participant in this revolution. It was almost certainly the first open‐access journal in radiology and one of the first in medicine. If you know of an earlier open‐access radiology journal, please send me the documentation.

Many younger medical physicists published their first article in the *JACMP* and have loyalty to it. They marketed themselves by linking their webpage CVs to the articles they published knowing there would be no barriers or copyright issues and that the entire article would be available to a potential employer. Today it is not unusual for a faculty physicist to earn tenure with up to half of the articles having been published in the *JACMP*. While 13 years ago there was a lot of doubt and more than a few naysayers, today medical physicists expect the *JACMP* to exist as an open‐source, open‐access journal. But when information wants to be free, who is going to pay the bills? Indeed, who pays the bills and who profits are essential questions to consider respecting the meaning of academic publishing.

Next issue, I intend to discuss the business model of an open‐access journal, and provide some tentative opinions as to how the bills might be paid and who should bear the cost. I invite you to send me your thoughts and ideas.

Michael D. Mills

Editor‐in‐Chief

January 16, 2013

